# Distinct unfolded protein responses mitigate or mediate effects of nonlethal deprivation of *C. elegans* sleep in different tissues

**DOI:** 10.1186/s12915-017-0407-1

**Published:** 2017-08-28

**Authors:** Jarred Sanders, Monika Scholz, Ilaria Merutka, David Biron

**Affiliations:** 1 Genetics, Genomics, and Systems Biology, The University of Chicago, Chicago, IL 60637 USA; 2 Institute for Biophysical Dynamics, The University of Chicago, Chicago, IL 60637 USA; 3 Department of Physics, The University of Chicago, Chicago, IL 60637 USA

**Keywords:** Sleep, *C. elegans*, Lethargus, Worm sleep, Unfolded protein response, Proteotoxicity, Proteostasis, Mitochondrial UPR, Endoplasmic reticulum UPR

## Abstract

**Background:**

Disrupting sleep during development leads to lasting deficits in chordates and arthropods. To address lasting impacts of sleep deprivation in *Caenorhabditis elegans*, we established a nonlethal deprivation protocol.

**Results:**

Deprivation triggered protective insulin-like signaling and two unfolded protein responses (UPRs): the mitochondrial (UPR^mt^) and the endoplasmic reticulum (UPR^ER^) responses. While the latter is known to be triggered by sleep deprivation in rodent and insect brains, the former was not strongly associated with sleep deprivation previously. We show that deprivation results in a feeding defect when the UPR^mt^ is deficient and in UPR^ER^-dependent germ cell apoptosis. In addition, when the UPR^ER^ is deficient, deprivation causes excess twitching in vulval muscles, mirroring a trend caused by loss of egg-laying command neurons.

**Conclusions:**

These data show that nonlethal deprivation of *C. elegans* sleep causes proteotoxic stress. Unless mitigated, distinct types of deprivation-induced proteotoxicity can lead to anatomically and genetically separable lasting defects. The relative importance of different UPRs post-deprivation likely reflects functional, developmental, and genetic differences between the respective tissues and circuits.

**Electronic supplementary material:**

The online version of this article (doi:10.1186/s12915-017-0407-1) contains supplementary material, which is available to authorized users.

## Background

Disrupting mammalian sleep during development correlates with negative effects on physical, cognitive, and social health, suggesting that sleep is important for appropriate development [1–3]. Nonlethal sleep deprivation also causes lasting neurological and behavioral deficits in *Drosophila melanogaster* [4]. However, a mechanistic grasp of why inadequate sleep during development is particularly deleterious is lacking.

*Caenorhabditis elegans* exhibits developmentally timed sleep during lethargus, a 2- to 3-h-long period at the termination of each larval stage [5–7]. Similar to mammalian sleep, lethargus is characterized by locomotion and feeding quiescence, sensory gating, a typical posture, rebound sleep, and deeply conserved regulation [6, 8–15].

Severe sleep deprivation activates DAF-16/FoxO, the *C. elegans* Forkhead box O (FoxO) transcription factor. FoxOs function broadly in regulating metabolism, lifespan, and responses to environmental stressors [16–24]. Nuclear translocation of DAF-16/FoxO is inhibited by the insulin/insulin-like growth factor signaling (IIS) pathway [17, 18]. In response to prolonged and continuous sleep deprivation, DAF-16 translocates to the nucleus to mitigate or delay lethality [10]. DAF-16 is also required for rebound sleep following much weaker disruptions [11].

A particular proteotoxic outcome of sleep deprivation is endoplasmic reticulum (ER) stress. In all species examined, including rodents and flies, expression of the ER chaperone immunoglobulin binding protein (BiP/Grp78) from the heat shock 70 protein family is upregulated upon sleep deprivation [25–30]. The *C. elegans* homolog of mammalian BiP is HSP-4 [31–33]. BiP/HSP-4 upregulation requires the action of the ribonuclease inositol-requiring protein-1 (IRE-1), a key receptor for sensing misfolded ER proteins [32–37]. IRE-1 signaling activates the XBP-1 transcription factor, which changes the expression of BiP and other genes in the deeply conserved unfolded protein response (UPR^ER^) pathway [25, 28, 30, 38–41].

Prolonged wakefulness increases daily energy expenditure [42–44], for instance, in the brain [45, 46]. Consequently, energy production by the mitochondrial oxidative phosphorylation system is upregulated [25, 30, 47–50]. Nevertheless, the roles of the mitochondrial UPR (UPR^mt^) following sleep deprivation are largely unknown, although one study found that sleep deprivation induces mitochondrial chaperones (to a lesser degree than BiP) in rat cerebral cortexes [30].

Upon mitochondrial stress, expression of *ubl-5*, encoding a ubiquitin-like protein, is upregulated, and UBL-5 plays a key role in activating dedicated chaperones and proteases of the UPR^mt^ [51–54]. In *C. elegans*, chemically induced mitochondrial stress upregulates the mitochondrion-specific chaperones HSP-6 and HSP-60 (from the Hsp70 and Hsp10/16 superfamilies) [51, 55].

Tractable model organisms have been prominently used to study responses to environmental stressors, such as oxidation or heat [56, 57]. In contrast, *C. elegans* sleep deprivation was minimally explored, and its lasting impacts, other than lethality, were never characterized. Here we established an automated approach to inflicting severe yet nonlethal deprivation of developmentally timed sleep in *C. elegans*. We found that worm sleep deprivation inflicts both mitochondrial and ER stress, as indicated by the triggering of the UPR^mt^ and the UPR^ER^. When the UPRs were genetically impeded, lasting defects in feeding, fecundity, and egg-laying physiology were detected. Moreover, different UPRs protected different tissues from the impacts of deprivation.

To assay feeding, we measured the pumping motion of the pharynx, a neuromuscular organ that takes in bacterial food, expels excess liquid, and passes food to the intestine [58, 59]. Maintaining these functions requires speed and regularity and is energetically costly. The pharyngeal nervous system consists of 20 neurons, is isolated from the rest of the animal by the basal lamina, and can operate independently [60, 61]. The rate of pumping depends on feeding history, quality of food, and endogenous serotonin levels [62–65]. In addition, pumping can be stimulated with exogenous serotonin [66, 67]. We found that sleep deprivation-induced mitochondrial stress impacts pharyngeal neurons and slows pumping.

In contrast, sleep deprivation-induced ER stress resulted in germ cell apoptosis and abnormal activity in the egg-laying circuit. The key determinant of *C. elegans* brood size is the number of available sperm [68]. Germ cell apoptosis can be triggered to protect sperm against DNA damage or environmental stressors that are not directly genotoxic. In both cases, highly conserved core apoptotic genes are strictly required for the initiation of programmed cell death [69–73]. One of these is CED-3, a cysteine-aspartate protease essential for execution of apoptosis [74–79]. Conveniently, this process can be visualized: the transmembrane receptor CED-1 mediates engulfment of early apoptotic corpses by surrounding sheath cells [80, 81]. Thus, the translational *ced-1::gfp* reporter is used to indicate the occurrence of germ cell apoptosis [82].

In addition, the egg-laying circuit exhibited a post-deprivation defect similar to the outcome of genetically ablating an egg-laying command neuron [83]. Opposite to the case of the feeding circuit, the UPR^ER^ (but not the UPR^mt^) mitigated the impact of deprivation in the egg-laying circuit. Collectively, these findings implicate two UPRs and insulin/insulin-like growth factor signaling in mitigating the impacts of disrupting worm sleep. They show that developmentally timed sleep is a vulnerable period: external stimuli that are benign outside of lethargus are proteotoxic when administered during lethargus. Adequate sleep promotes normal functions in tissues differing in developmental dynamics and physiological activity, and distinct UPRs mitigate different impacts of nonlethal deprivation.

## Results

### Substantial deprivation of lethargus quiescence can be automatically inflicted

**Fig. 1:**
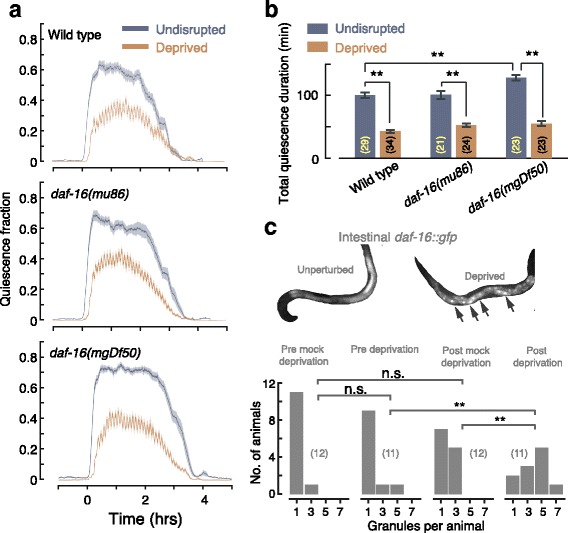
A periodic mechanical stimulus can partially and nonlethally reduce lethargus quiescence and induce translocation of DAF-16. **a** The fraction of quiescence measured during L4 lethargus of undisrupted (*gray*) and deprived (*orange*) wild-type animals and *daf-16* mutants. Locomotion was forced using a square wave of mechanical vibrations with a 6-min period and 50% duty cycle. *N* = 30 animals, *shaded areas* depict mean ± standard error of the mean (*SEM*). **b** The total time in which quiescence was observed integrated over the data presented in **a**. Error bars depict mean ± SEM. **c** DAF-16::GFP fluorescence in the intestine before and after 1 h of sleep deprivation. *Top*: examples of green fluorescent protein (*GFP*) fluorescence in unperturbed and partially deprived animals. *Arrowheads* point to bright particles indicating concentration of DAF-16::GFP. *Bottom*: Histograms of the number of bright particles per animal identified under each set of conditions. Sample sizes are denoted in parentheses. *Single* and *double asterisks* denote significant differences with *p* < 0.05 and *p* < 0.01, respectively

Forced locomotion inflicted during *C. elegans* sleep by manually delivering harsh touch was previously shown to be lethal [6, 10]. Considerably more gentle mechanical vibrations can transiently force motion. We previously identified rebound sleep when vibrations were applied infrequently [11] and found that worms desensitized to vibrations delivered too frequently. In response to a 3-min on/off cycle of vibrations, we measured a 50% reduction in mean quiescence (Fig. 1a, b). This indicated that, in contrast to previously published conditions, the 3-min on/off cycle robustly overwhelmed the capacity of the worms to compensate for excess motion. We further found that this disruption affected wild-type animals and *daf-16* mutants similarly.

Manually forced locomotion during worm sleep drives translocation of DAF-16 to nuclei of intestinal and body wall muscle cells [10]. We asked whether our 3-min on/off disruptions would induce similar translocation of DAF-16. To address this, we exposed animals expressing fluorescently labeled DAF-16 to 1 h of the vibration stimuli during the first half of the fourth lethargus stage (L4 lethargus). In agreement with the manual (lethal) deprivation protocol, we observed nuclear localization in intestinal cells (Fig. 1c). Translocation was not observed following a mock perturbation protocol, where vibrations were not applied during an equivalent 1-h period. In our hands, a clear translocation response was not observed in body wall muscles.

In contrast to the consequences of continuous manual deprivation [10], we did not observe any molting defects or lethality following our deprivation conditions. Possibly, this was a consequence of not depriving the animals of quiescence for a continuous period that exceeded 3 min. Collectively, these data demonstrate the ability to automatically and severely disrupt quiescence to a stressful yet nonlethal degree.

Considering these results, the experiments described throughout the manuscript employ three types of deprivation conditions: 1 h of disruptions for acute responses in individual animals expressing fluorescent markers (“mock” animals were loaded to identical observation chambers), 4 h of disruptions for gene expression assays in small groups of tightly synchronized animals, and 12 h of disruptions for lasting effects of deprivation assayed in large groups of animals. To control for nonspecific effects of the prolonged stimulation period, we compared sleep-deprived animals to those exposed to vibrations outside of lethargus. These groups were labeled “control” (see Methods section for details). In the latter two protocols (4 h and 12 h), sleep was disrupted for no more than the 3-h duration of lethargus. To identify effects of vibrations that are nonspecific to lethargus, we assayed a subset of strains without exposing them to any vibrations. These groups were labeled “unperturbed,” and they consistently exhibited similar phenotypes to those of the “control” animals. Thus, nonspecific effects of vibrations were found to be minor and genetically separable from impacts of sleep deprivation.

### Two unfolded protein responses are triggered by nonlethal deprivation of *C. elegans* sleep

**Fig. 2:**
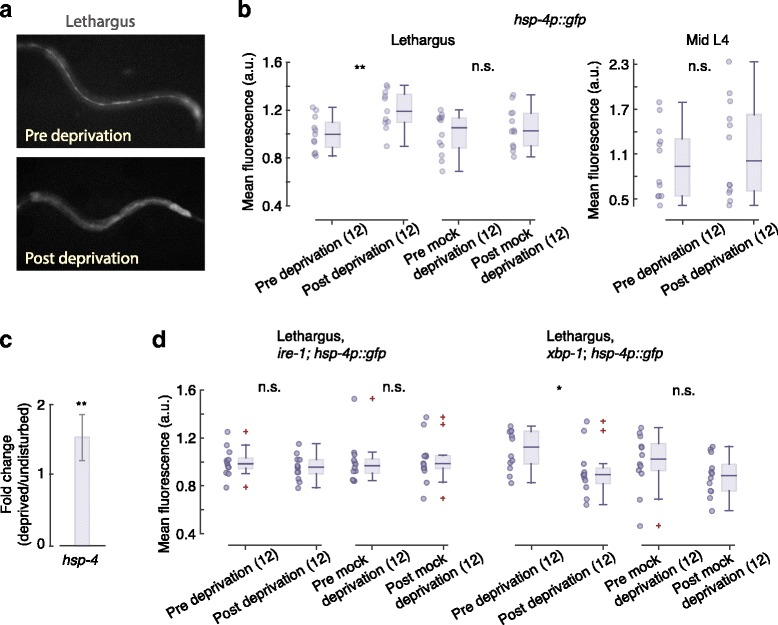
Worm sleep deprivation triggers the UPR^ER^. **a** Example of pre- (*top*) and post- (*bottom*) deprivation fluorescence of the *hsp-4p::gfp* reporter. Prior to deprivation the reporter was prominently observed in the seam cells. **b** Fluorescence of the *hsp-4p::gfp* reporter before and after deprivation, mock deprivation, and stimulation of mid-L4 larvae. **c** Quantification of *hsp-4* expression using real-time PCR. Error bars depict 99% confidence intervals (1 biological replicate, 20 animals per sample). **d** Fluorescence of the *hsp-4p::gfp* reporter in mutants where the function of the UPR^ER^ genes *ire-1* and *xbp-1* was lost. On these mutant backgrounds sleep deprivation did not upregulate the expression of *hsp-4*. In all box plots, *horizontal lines*, *boxes*, and *bars* depict medians, 1^st^ and 3^rd^ quartiles, and 5th and 95th percentiles, respectively. *Crosses* denote outliers. *Single* and *double asterisks* denote significant differences with *p* < 0.05 and *p* < 0.01, respectively

DAF-16/FoxO is associated with a broad spectrum of stress responses, and ER stress in particular was detected in previously examined sleep-deprived animals [25, 38–40]. To address whether the UPR^ER^ was triggered by worm sleep deprivation, we used a transcriptional reporter for HSP-4/BiP expression, *hsp-4p∷gfp (zcIs4)* [33, 51]. Animals expressing this indicator were subjected to a 1-h deprivation protocol. During L4 lethargus, *hsp-4* was notably expressed in the epithelial seam of undisrupted animals (Fig. 2a). This array of hypodermal stem cells, termed seam cells, regulate hypodermal/cuticle formation and transform to their adult fate at the time of the fourth molt [7, 84–86]. We observed that *hsp-4* expression in seam cells coincided with the generation of the alae — the adult cuticular ridges.

Neither mock deprivation nor 1 h of vibration stimuli prior to lethargus affected *hsp-4* expression. In contrast, sleep-deprived animals significantly upregulated the expression of *hsp-4* (Fig. 2b). Consistently, *hsp-4* expression remained elevated after a 4-h period of disruptions, as indicated by quantitative PCR (Fig 2c). Upregulation of *hsp-4* requires essential components of the UPR^ER^ including IRE-1 and XBP-1 [33, 34, 87]. Consistent with induction of ER stress by environmental stressors or genetic perturbations, sleep deprivation failed to upregulate *hsp-4* expression on *ire-1* or *xbp-1* mutant backgrounds (Fig. 2d).

**Fig. 3:**
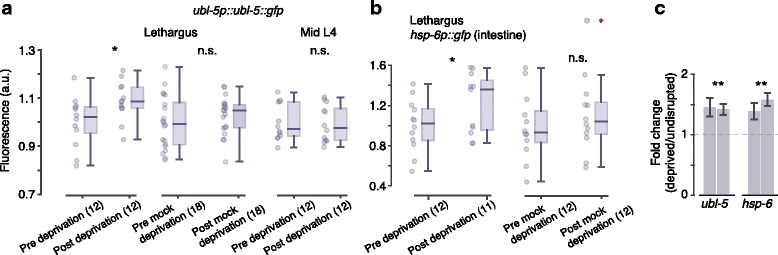
Worm sleep deprivation triggers the UPR^mt^. **a** Intestinal fluorescence of the *ubl-5p::ubl-5::gfp* UPR^mt^ reporter before and after deprivation, mock deprivation, and stimulation of mid-L4 larvae. **b** Intestinal fluorescence of the mitochondrial chaperone *hsp-6p::gfp* reporter after deprivation and mock deprivation. *Horizontal lines*, *boxes*, and *bars* depict medians, 1^st^ and 3^rd^ quartiles, and 5th and 95th percentiles, respectively. *Single* and *double* asterisks denote significant differences with *p* < 0.05 and *p* < 0.01, respectively. **c** Relative expression of *ubl-5* and *hsp-6* in deprived as compared to undisrupted wild-type animals (2 biological replicates, 20 animals per sample). The mechanical stimulus was applied for 4 h (which included L4 lethargus), and RNA was prepared immediately after this period. Error bars depict mean ± SEM

The roles of the UPR^mt^ following sleep deprivation are less understood, although evidence of mitochondrial stress was detected in sleep-deprived rodents [30]. Therefore, we similarly used fluorescent indicators to assay the activation of the UPR^mt^ [51–53]. The *ubl-5* translational reporter of the UPR^mt^ expresses broadly at low levels and brightly in the posterior bulb of the pharynx, the posterior of the intestine, and the anterior edge of the intestine near the pharyngeal-intestinal valve. Following 1 h of sleep deprivation, we observed a small but significant upregulation of intestinal *Publ-5::ubl-5::gfp* expression. No increase in reporter fluorescence was observed following the mock protocol or when vibrations were applied at the mid-L4 larval stage (Fig. 3a).

We similarly assayed two additional indicators of the UPR^mt^: the *hsp-6* and *hsp-60* transcriptional reporters. Expression of *hsp-6* was broad and most clearly visible in the intestine. Strong expression of *hsp-6* or an accumulation of the reporter led to bright staining of a posterior segment of the intestine. Sleep deprivation did not affect the posterior bright patch, but expression in the rest of the intestine was upregulated after the 1-h deprivation protocol. Mock deprivation did not affect *hsp-6* expression (Fig. 3b). Real-time PCR was used to assay the relative expression of these genes after a 4-h period of administering the disruptive stimuli that included L4 lethargus. Consistently, we observed elevated expression of *ubl-5* and *hsp-6* in deprived animals (Fig. 3c). In our hands, we could not detect upregulation of *hsp-60* post-deprivation (Additional file 1: Figure S1). Combined, these results show that nonlethal deprivation of worm sleep is proteotoxic and induces both mitochondrial and ER stress. Comparable stimulation outside of lethargus did not trigger these UPRs, indicating that the period of sleep is particularly vulnerable.

### The UPR^mt^ plays a role in mitigating effects of nonlethal sleep deprivation on pumping

Pharyngeal pumping for the purpose of feeding is an energetically demanding behavior that can readily be quantified [65]. To identify lasting effects of sleep deprivation on pumping, each animal was assayed for 60 min at a food concentration corresponding to an optical density (OD_600_) = 2.5 of the bacterial suspension, where pumping activity was intermediate (Additional file 2: Figure S2). Since control and unperturbed animals (as defined above) exhibited nearly identical feeding behaviors, deprivation conditions were typically compared to stimulation outside of lethargus (control).

**Fig. 4:**
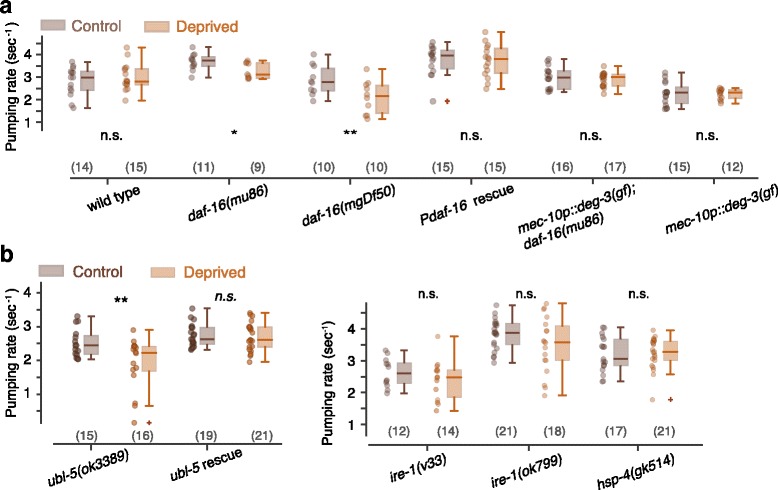
Mitigating mitochondrial (but not ER) stress is required for maintaining normal post-deprivation pumping rates. **a** Box plots of average pumping rates for control (i.e., stimulated outside of lethargus) and deprived animals obtained with the optical tracking method. Loss of DAF-16 or *mec-10* expressing neurons confers a broad defect in stress responses or the loss of gentle mechanosensation, respectively. **b** Average pumping rates of animals deficient in the UPR^mt^ (*left*) and the UPR^ER^ (*right*). *Horizontal lines*, *boxes*, and *bars* depict medians, 1^st^ and 3^rd^ quartiles, and 5th and 95th percentiles, respectively. Sample sizes are noted in parentheses; *asterisks* and *double asterisks* denote significant differences (*p* < 0.05 and *p* < 0.01, respectively)

Pharyngeal pumping can be adequately described as bursts of rapid pumping interspersed with pauses [65]. Similar to a previous study, we measured the mean instantaneous pumping rate and the duty ratio of rapid pumping [88]. These summary statistics did not reveal significant differences between deprived and control wild-type animals. However, sleep deprivation reduced pumping in *daf-16* mutants, and the native promoter rescue of DAF-16 function restored the wild-type phenotype (Fig. 4a and Additional file 3: Figure S3). These results suggest that worm sleep deprivation can negatively impact feeding in a DAF-16-dependent manner.

To control for potential biomechanical impacts of the vibrations, we assayed worms expressing *deg-3(u662)*, a degeneration-causing constitutively active nicotinic acetylcholine receptor (nAChR) channel subunit, in touch neurons expressing the *mec-10* gene [89–93]. These transgenics did not respond to vibrations (Additional file 4: Figure S4). In the absence of *mec-10* expressing neurons, vibration stimuli during lethargus did not affect subsequent pumping on both wild-type and *daf-16* mutant backgrounds (Fig. 4a and Additional file 3: Figure S3). These data indicate that mechanosensation by *mec-10* expressing neurons and the ensuing loss of quiescence are required for pumping fatigue following sleep deprivation.

To test whether the UPR^mt^ plays a role in mitigating consequences of nonlethal sleep deprivation, we examined feeding in *ubl-5* mutants. Deprived *ubl-5* mutants exhibited a decrease in pumping rate as compared to the control group. This pumping defect was rescued by expressing the *Publ-5::ubl-5::gfp* translational reporter (Fig. 4b and Additional file 3: Figure S3). In contrast, UPR^ER^ defective worms did not exhibit pumping fatigue: animals carrying two *ire-1* putative null alleles and *hsp-4* mutants did not significantly change their mean pumping rate post-deprivation (Fig. 4b). A mild change in the duty ratio of deprived *ire-1* mutants may indicate a weak contribution of the UPR^ER^ maintaining post-deprivation pumping (Additional file 3: Figure S3). These results demonstrate that ER proteotoxic stress, unlike mitochondrial stress, does not play a major role in mitigating pumping fatigue following sleep deprivation.

Complementarily, electropharyngeograms (EPGs) enabled us to precisely time contractions and relaxations of the pharyngeal corpus and terminal bulb. We therefore used EPGs to measure durations of individual pumps and to confirm our optical measurements of pumping rates (Additional file 5: Figure S5, Additional file 6: Figure S6, and Additional file 7: Figure S7). We found that repeated mechanical stimuli extended the duration of individual pumps. However, the extension of single pumps was not specific to sleep deprivation; deprived animals and controls exposed to the mechanical stimuli before and after lethargus exhibited similar pump durations. The extended duration of individual pumps in *ubl-5* mutants was comparable to that of wild-type animals and *daf-16* mutants (Additional file 6: Figure S6). Thus, we could differentiate between deprivation-related and nonspecific impacts of mechanical stimuli, and we did not identify a role for the UPR^mt^ in mitigating the nonspecific effect. Combined, our results indicate that the UPR^mt^ (but not the UPR^ER^) mitigates lasting effects of sleep deprivation on pharyngeal pumping.

### Nonlethal sleep deprivation impacts pharyngeal pumping by affecting regulatory neurons

The pharynx is isolated from the rest of the animal and can exhibit pumping tens of minutes after it has been dissected out [60, 61]. Pumping defects induced by sleep deprivation can thus originate from pharyngeal regulatory neurons or pharyngeal muscles. We note that our mechanical stimuli do not noticeably affect the buccal plug, a cap of extracellular material that prevents food from entering the pharynx during lethargus [7], and they do not induce pumping. Consequently, the stimuli do not activate the pharyngeal muscles during lethargus, and “wear and tear” damage caused by anachronistic muscle activation is unlikely.

**Fig. 5:**
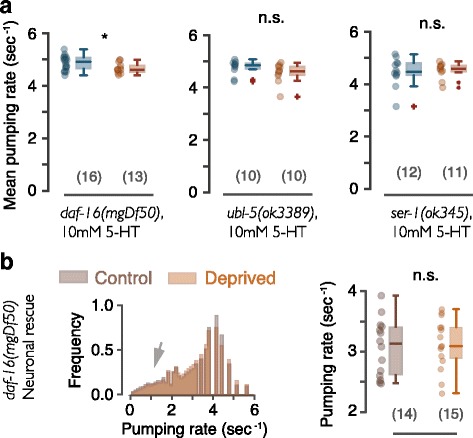
A neuronal deficiency underlies the post-deprivation slowdown of feeding. **a** Box plots of pumping rates for 5-HT-triggered pumping in undisrupted and deprived *daf-16(mgDf50)*, *ubl-5*, and *ser-1* mutants. **b** *Left*: distributions of pumping rates for deprived and control *daf-16* mutants where neuronal function of DAF-16 was restored. *Arrow* points to the indistinguishable left tails of the distributions, indicating similar statistics of sporadic pumping. *Right*: mean pumping rates under these conditions. *Horizontal lines*, *boxes*, and *bars* depict medians, 1^st^ and 3^rd^ quartiles, and 5th and 95th percentiles, respectively. Sample sizes are noted in parentheses; *asterisks* and *double asterisks* denote significant differences (*p* < 0.05 and *p* < 0.01, respectively)

Serotonin or 5-hydroxytryptamine (5-HT) robustly activates rapid pumping through the action of the neuronally expressed SER-7/5-HT7 receptor. Several labs have shown that 5-HT-induced rapid pumping is abolished in *ser-7* null mutants [64, 66, 67, 88]. We similarly activated pharyngeal neurons with 10 mM 5-HT instead of food and assayed pumping fatigue. We found that 5-HT-induced pumping was rapid in undisrupted and deprived *daf-16(mgDf50)* and *ubl-5* mutants (Fig. 5a).

An additional serotonin receptor, SER-1/5-HT2, is expressed in pharyngeal muscles [94] but is not required for rapid pumping, whether induced by food or by 5-HT [64, 95]. Consistently, mutants carrying the putative null allele *ser-1(ok345)* and treated with 5-HT exhibited high pumping rates and no pumping fatigue (Fig. 5a). The absence and presence of rapid 5-HT-induced pumping in *ser-7* and *ser-1* mutants, respectively, indicates that 5-HT acts through activating pharyngeal neurons. The ability of deprived *daf-16* and *ubl-5* mutants to pump rapidly suggests that their deficits, exhibited in the presence of food, are the result of regulation rather than a biomechanical defect. In addition, neuronal rescue of DAF-16 function abolished the post-deprivation phenotype (Fig. 5b). Combined, these data indicate that rapid pumping is mechanically possible even in sleep-deprived mutants upon activation of pharyngeal neurons. Thus, sleep deprivation likely leads to lasting deficits in the neural circuit regulating pumping.

### Nonlethal deprivation results in UPR^ER^-dependent reduction in brood size

**Fig. 6:**
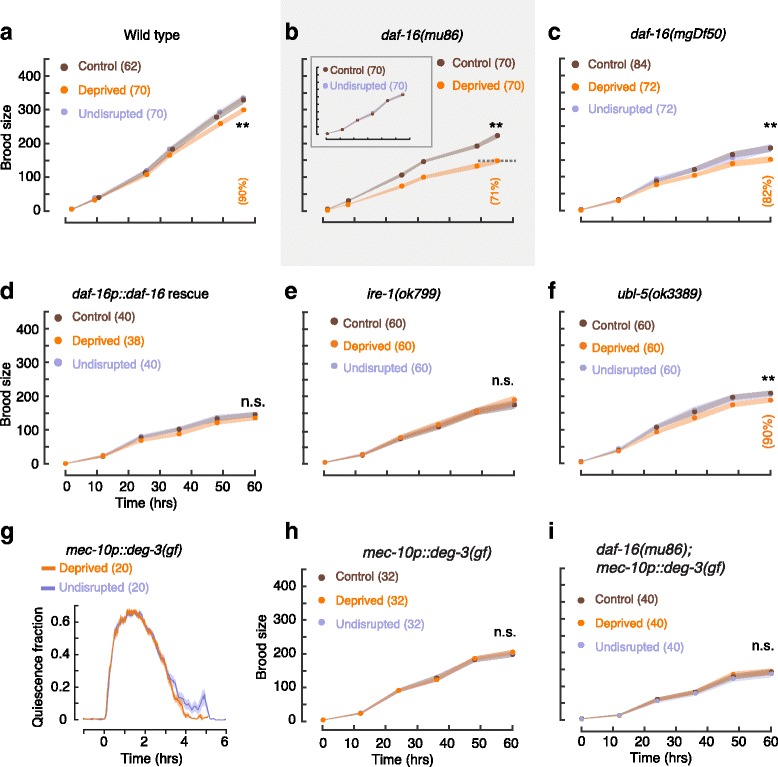
Worm sleep deprivation reduces brood size. **a** Brood sizes of wild-type animals during the first 3 days after L4 lethargus (*t* = 0 is 10–12 h after the fourth molt). Prior to the assay, animals were exposed to the 12-h deprivation protocol. The control group was stimulated ouside of lethargus, and the undisrupted group was not stimulated (see Methods). The *dotted line* depicts the brood size of deprived animals at the latest time point (see also Fig. 7a). *Inset*: undisrupted and control animals were indistinguishable. **b**, **c** The same as **a** for *daf-16(mu86)* and *daf-16(mgDf50)* mutants. **d** The same as **a** for *daf-16(mgDf50)* mutants where expression of *daf-16* cDNA was driven by the *daf-16* native promoter. **e**, **f** The same as **a** for UPR^ER^ deficient *ire-1* mutants and UPR^mt^ deficient *ubl-5* mutants. The wild-type phenotype was exhibited by *ubl-5* mutants, but fecundity was not reduced in *ire-1* mutants. **g** Quiescence fraction during L4 lethargus of animals deficient in touch sensation (*mec-10p::deg-3(gf)*), whose responses to vibrations were mostly or entirely abolished (see also Additional file 4: Figure S4). **h**, **i** The same as **a** for transgenic animals deficient for touch sensation on wild-type or *daf-16* mutant backgrounds. *Shaded areas* depict mean ± SEM, numbers of animals assayed are noted in parentheses, and *double asterisks* denote a significant difference in brood size at *t* = 60 h (*p* < 0.01)

Sleep deprivation was recently implicated in affecting fertility in rodents [96, 97]. To address whether nonlethal sleep deprivation impacts *C. elegans* fecundity, we compared brood sizes of deprived and control animals using the 12-h protocol. We found that brood size in the control group was indistinguishable from that of undisrupted animals. However, sleep deprivation reduced wild-type brood size by 10% (Fig. 6a). The negative impact of nonlethal deprivation was exacerbated in *daf-16(mu86)* mutants, where brood size was reduced by 29% (Fig. 6b). In *daf-16(mgDf50)* null mutants, where brood size was overall lower, we observed an 18% reduction (Fig. 6c). When the function of DAF-16 was restored by driving expression with its native promoter, brood size was not reduced following deprivation (Fig. 6d).

To address the possibility of a floor effect in the *daf-16(mgDf50)* animals, we increased the brood size of *daf-16(mgDf50)* mutants through male mating [68] and observed a 24% reduction in brood size when both hermaphrodites and males were deprived. Disrupting sleep of either hermaphrodites or males resulted in intermediate phenotypes (Additional file 8: Figure S8A).

Since germ cell apoptosis can be enhanced by pharmacologically or genetically induced ER stress [98], we hypothesized that the UPR^ER^ may mediate an adverse impact of sleep deprivation on fecundity. To test this, we compared *ire-1* mutants deficient in the UPR^ER^ to wild-type animals, *daf-16* mutants, and UPR^mt^ deficient *ubl-5* mutants. In contrast to the other genotypes, where deprivation reduced fecundity, brood size was not reduced in the absence of IRE-1. Rather, deprived, control, and unperturbed *ire-1* mutants were indistinguishable (Fig. 6e). Similarly, fecundity was impervious to sleep deprivation when ASI neurons, required for UPR^ER^-mediated germ cell apoptosis, were genetically ablated (Additional file 8: Figure S8B) [98, 99]. The wild-type phenotype exhibited by *ubl-5* mutants served as a negative control and indicated that the UPR^mt^ did not mediate the impact of deprivation on brood size (Fig. 6f).

To control for potential biomechanical effects of the vibrations, we assayed *deg-3(u662)* transgenics. Failure to respond to vibrations abolished the reduction in brood size in the presence of the stimuli (Fig. 6h, i). Forced locomotion also did not appreciably increase the number of retained eggs (Additional file 9: Figure S9). Overall, these results suggest that nonlethal sleep deprivation in *C. elegans* negatively impacts brood size and that the UPR^ER^ and IIS play distinct roles in this process. Together with the relevance of the UPR^mt^ to pumping fatigue, these data demonstrate distinct UPRs for mitigating impacts of sleep deprivation in different tissues.

### Germ cell apoptosis causes the brood size reduction following nonlethal deprivation

**Fig. 7:**
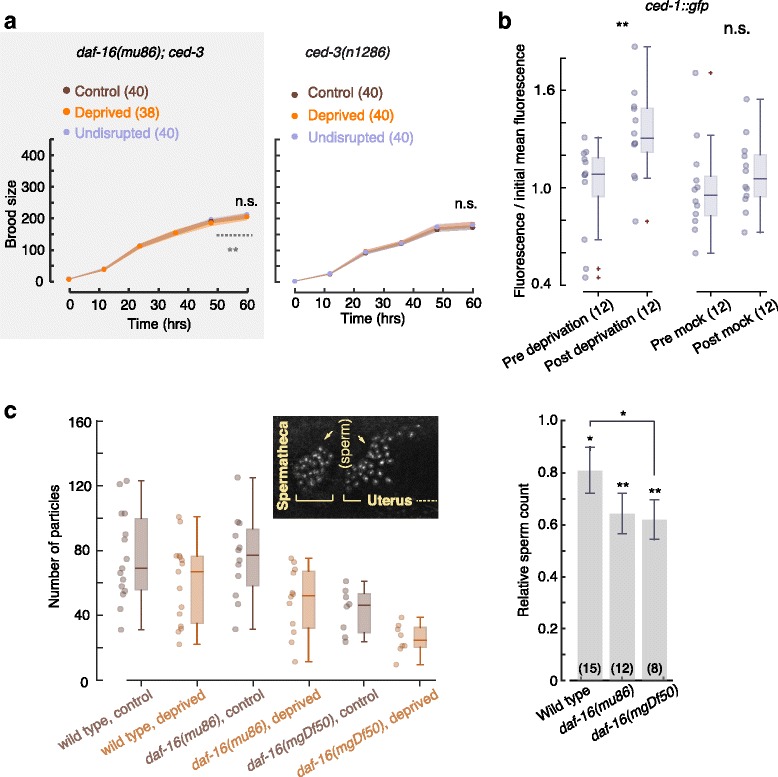
Worm sleep deprivation causes germ cell apoptosis. **a** Brood sizes of *daf-16(mu86); ced-3(n1286)* double mutants during the first 3 days after L4 lethargus (*t* = 0 is 10–12 h after the fourth molt). Deprivation failed to induce a reduction in brood size. The *dotted line* depicts the brood size of deprived *daf-16(mu86)* mutants at *t* = 60 h from Fig. 6a. **b** Box plots of *ced-1::gfp* fluorescence after deprivation (*left*) or mock deprivation (*right*), normalized by the mean pre-treatment fluorescence. *Horizontal lines*, *boxes*, and *bars* depict medians, 1^st^ and 3^rd^ quartiles, and 5th and 95th percentiles, respectively. Sample sizes are denoted in parentheses; *double asterisks* depict a significant difference (*p* < 0.01). **c** *Left*: the number of sperm detected in a single gonad per animal. *Horizontal lines*, *boxes*, and *bars* depict means, 95% confidence intervals, and standard deviations, respectively. *Inset*: a confocal image of adult hermaphrodite sperm nuclei, specifically labeled by GFP-histone fusion driven by the *Pcomp-1* promoter. *Right*: the ratio between the sperm count of deprived and control animals. Error bars depict mean ± SEM. Sample sizes are denoted in parentheses; *single* and *double asterisks* depict significant differences (between the mean and 1 or between genotypes) with *p* < 0.05 or *p* < 0.01, respectively

The requirement of the UPR^ER^ for the reduction in brood size suggested that sleep deprivation may trigger germ cell apoptosis [98]. To test this, we assayed mutants lacking core apoptotic machinery caspase, quantified a fluorescent indicator of germ cell apoptosis, and counted fluorescently labeled germ cells. Animals carrying the putative null allele *ced-3(n1286)* were crossed to *daf-16(mu86)* mutants, which exhibited the most pronounced effect on fecundity. In both *ced-3* single and *daf-16; ced-3* double mutants the post-deprivation reduction in brood size was eliminated; i.e., the egg-laying dynamics of deprived, control, and unperturbed animals were indistinguishable (Fig. 7a). Moreover, brood sizes of *daf-16; ced-3* double mutants were identical to those of undisrupted *daf-16(mu86)* single mutants. This suggested that the *ced-3* mutation did not affect fecundity independently of sleep deprivation on a *daf-16* background.

The *ced-1::gfp* translational reporter is an established indicator of germ cell apoptosis [82]. Animals expressing this reporter were subjected to the 1-h deprivation protocol. Deprivation resulted in a significant increase in *ced-1::gfp* fluorescence, suggesting that germ cell corpses were actively being disposed of. In contrast, the mock deprivation group, i.e., worms not exposed to vibration stimuli, did not exhibit a change in reporter fluorescence during the equivalent period (Fig. 7b).

Next, we counted fluorescently labeled sperm in self-fertilized deprived and control hermaphrodites as previously described [100, 101]. We observed reductions in sperm count following forced locomotion during L4 lethargus as compared to the control groups in wild-type and the two *daf-16* strains (Fig. 7c). The reduced sperm counts were sufficient to explain the corresponding reductions in brood sizes. The reductions in *daf-16(mgDf50)* and *daf-16(mu86)* mutants were similar, perhaps due to the variability of the measurement, the 3-day assay having fallen short of revealing the full extent of the brood size deficiency, or further loss of sperm during adulthood. Direct indication of germ cell corpse engulfment mediated by CED-1, the requirement of the caspase CED-3, and the reduced sperm count provide three consistent lines of evidence. Together, they show that sleep deprivation induces germ cell apoptosis, consistent with the role of the UPR^ER^ in brood size reduction.

### The UPR^ER^ (but not the UPR^mt^) mitigates the effects of nonlethal sleep deprivation on activity in the egg-laying circuit

**Fig. 8:**
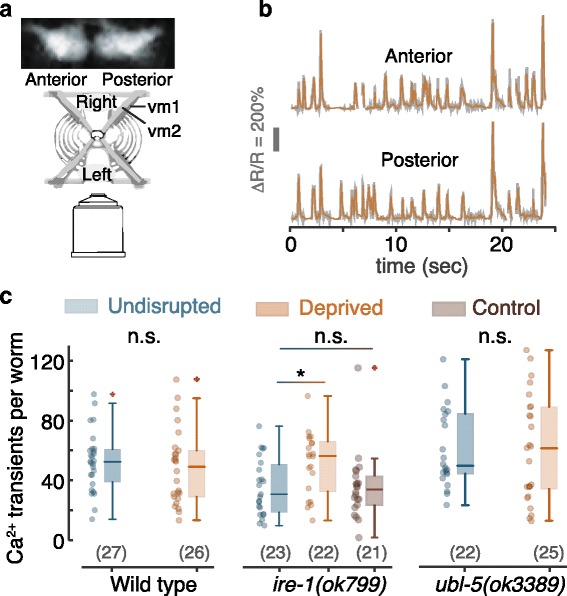
Worm sleep deprivation results in excess twitching of vulval muscles. **a** A schematic representation of the imaging setup. *Top*: fluorescently labeled anterior and posterior vulval muscle groups. Worms crawl on their left or right side such that their dorsoventral axis is parallel to the imaging plane. **b** Example traces of the ratio of GCaMP5 to mCherry fluorescence from anterior and posterior vms. **c** The total number of calcium transients in vms of undisrupted and sleep-deprived worms. An average increase of 30% in the number of vm twitches was detected in *ire-1* (UPR^ER^ deficient) mutants. No significant changes were detected in wild-type animals and *ubl-5* (UPR^mt^ deficient) mutants. *Horizontal lines*, *boxes*, and *bars* depict medians, 1^st^ and 3^rd^ quartiles, and 5th and 95th percentiles, respectively. Sample sizes are noted in parentheses, and *the asterisk* denotes a significant difference (*p* < 0.02)

The absence of hermaphrodite-specific neurons (HSNs) suppresses egg laying and doubles the frequency of calcium transients in vulval muscles (vms) [83, 102]. As germ cell apoptosis does not preclude an independent impact of deprivation on the egg-laying circuit, we asked whether sleep deprivation can cause abnormally high calcium activity in the vms. To address this, we assayed calcium dynamics indicative of vm twitching using a ratiometric reporter: co-expression of the calcium indicator GCaMP5 and the red fluorescent protein mCherry in the vms [83, 103] (Fig. 8a, b).

Opposite to the pharyngeal circuit, the egg-laying circuit was affected by ER but not mitochondrial stress. Neither wild-type animals nor UPR^mt^ deficient *ubl-5* mutants exhibited abnormal post-deprivation activity. When UPR^ER^ deficient *ire-1* mutants were deprived, their mean number of vm twitches increased by 30%, mirroring the trend reported for HSN deficient animals (Fig. 8c).

Physiological activity in the egg-laying circuit is coupled with body posture and locomotion during brief periods around egg-laying events [83, 103, 104]. Therefore, we could not rule out the possibility that differences in locomotion over long timescales may also correlate with activity in the egg-laying circuit. Specifically, we asked whether a potential effect of deprivation on locomotion may indirectly cause the vm twitching phenotype observed in *ire-1* mutants. To address this, we assayed the effect of sleep deprivation on mean velocity and measured the correlations between mean velocity and physiological activity (twitches) in the vms.

In all genotypes assayed, the mean velocity did not vary significantly between undisrupted and deprived animals (Additional file 10: Figure S10). While correlations between the mean velocity and vm twitching were found in wild-type, UPR^ER^ deficient, and UPR^mt^ deficient worms, they were weakest in *ire-1* mutants (Additional file 11: Figure S11). Moreover, the differences between mean or median velocities of deprived and nondeprived worms were insufficient to account for the 30% increase in vm twitching. Thus, excess vm twitching was not an indirect consequence of elevated locomotion activity in our assays.

Interestingly, the correlations between activity in the egg-laying circuit and locomotion were stronger in deprived wild-type animals and *ubl-5* mutants as compared to their respective undisrupted groups. This trend was reversed in *ire-1* mutants (Additional file 11: Figure S11). This observation may indicate that elevated coordination between distinct behaviors during stress may require secreted proteins, such as neuropeptides, whose function depends on processing in the ER [105]. These results support the conclusion that sleep deprivation causes ER stress which, if not mitigated, impacts the egg-laying circuit. Taken together, our data demonstrate that distinct types of proteotoxic stress caused by nonlethal deprivation of worm sleep adversely affected different circuits.

## Discussion

The cognitive, physiological, and behavioral changes resulting from deprivation of human sleep can be subtle and elude superficial phenotyping. Detecting them requires functional imaging and/or proper design of the task being assayed, a clear definition of the sleep deprivation conditions, and careful measurements. Interpreting such results involves consideration of details such as differences in vulnerability to deprivation between individuals and whether the task was monotonous or complex [106–109]. Similarly, wild-type rodents and insects do not typically exhibit gross defects or substantial damage to brain cells following nonlethal deprivation protocols [4, 110–112]. This may be due to protective responses, activated by sleep deprivation, with the capacity to effectively prevent or repair the resulting damage.

To address the lasting impact of sleep deprivation in *C. elegans,* we established an experimental method enabling severe reduction in quiescence with no lethality or molting defects. Our periodic stimulus allowed for some quiescent behavior to take place throughout lethargus. Possibly, not forcing locomotion for extended continuous periods was key to avoiding a lethal outcome. In contrast, previous work assessed the impact of total sleep deprivation, i.e., consecutive forced movement for 30 min, which resulted in lethal molting defects. The impact on molting was interpreted to indicate a defect in metabolic regulation during lethargus, as the loss of DAF-16 sensitized the animals to this effect [10]. Lasting defects in surviving animals were not previously assayed.

The complete lack of lethality despite a loss of 50% of quiescence during lethargus suggests that quiescence, in and of itself, is an imperfect measure of the quality and restorative benefits of developmentally timed sleep in *C. elegans*. If homeostatic compensation can affect the quality of sleep [13], periodically allowing for rebound could confer greater restoration per unit time as compared to uninterrupted sleep. Testing this hypothesis will be key for understanding lethargus and may promote our understanding of additional quiescent states.

Identifying protective responses to worm sleep deprivation may clarify ancient functions of sleep. Cellular damage and repair can vary in molecular detail and occur at various rates. A comparison between tissues with different functions, developmental states, and metabolic demands may resolve requirements for a rest period due to accumulating damage. We found that deprivation triggers the UPR^mt^, which protects feeding behavior in sleep-deprived worms. In contrast to the UPR^ER^, the UPR^mt^ was not strongly associated with sleep deprivation previously. This may be partly due to a focus on sleep-related changes in gene expression in the brain [25, 27, 28, 38–40, 113–116]. One study reported that the mitochondrial chaperones Hsp60 and glucose-regulated protein 75 (Grp75, from the Hsp70 superfamily) were upregulated in the cerebral cortex of rats after sleep deprivation, although not as much as BiP [30].

Broad transcriptional responses to sleep deprivation were reported in mouse livers, lungs, and hearts [117, 118]. These studies demonstrate that the molecular consequences of disrupting sleep are not limited to the brain and that sleep contributes to normal function in a manner that may vary between different tissues or organs. For instance, the pharynx of *C. elegans* is a highly active organ, required to repeatedly generate powerful contractions. Neurons that regulate pumping may face a distinct cellular metabolic challenge and therefore may particularly benefit from the UPR^mt^ post-deprivation.

Nonlethal sleep deprivation upregulates the expression of BiP in rodents and flies, thus indicating the activation of the UPR^ER^ [25, 38–40]. We have shown a similar upregulation of HSP-4/BiP in response to disrupting developmentally timed worm sleep. Furthermore, loss of function of the misfolded ER protein receptor IRE-1 affected both fecundity and the egg-laying circuit post-deprivation. These findings in *C. elegans* show that the activation of the UPR^ER^ is a deeply conserved response to sleep deprivation [15]. It remains to be determined whether the complementing set of phenotypes exhibited by the pharyngeal and the egg-laying circuits, whose function depended on the UPR^mt^ and the UPR^ER^, respectively, is associated with differences in their developmental states, energy expenditure, or additional factors.

Finally, we characterized outcomes of nonlethal sleep deprivation with respect to feeding, fecundity, and egg-laying physiology. Consistent with previous findings, our deprivation protocol triggered translocation of DAF-16/FoxO into intestinal cell nuclei [10] and implicated it in mitigating lasting adverse effects of deprivation. Three lines of evidence indicated that fecundity was reduced due to germ cell apoptosis: a dependence of the effect on CED-3 and IRE-1, a low sperm count in sleep-deprived worms, and the engulfment of early germ cell corpses by surrounding sheath cells as visualized by the *ced-1* reporter [74, 75, 81, 98]. Interestingly, sperm quality was recently shown to be negatively impacted by disruptions to sleep in rodents [96, 97].

## Conclusions

Hypotheses explaining the core functions of sleep include the notion that its utility may differ across species [119]. The synaptic homeostasis hypothesis proposes that continuous learning during wakefulness is bound to saturate synaptic connections. Therefore, renormalization of net synaptic strength during sleep is required to restore homeostasis [120, 121]. Other suggestions focus on “wear and tear” in physiologically active neurons during wakefulness due to accumulation of protein fragments, unfolded proteins, or other molecular stressors [112, 122–125]. On a larger scale, metabolite clearance from the brain can increase during mammalian sleep [126]. In addition, disrupting sleep is linked to abnormal glucose metabolism and appetite regulation. These findings suggest that sleep is key to normal metabolic and hormonal processes outside the brain [127, 128].

This work describes multiple pathways by which sleep deprivation can upset cellular proteostasis, likely by creating unfavorable biochemical conditions. Specific characteristics of physiological activity and development could affect the balance between accumulation and relief of allostatic load. To the best of our knowledge, how this balance might scale with different types of metabolic loads has not been systematically studied, let alone connected to sleep. Protection of post-deprivation pharyngeal function by the UPR^mt^ is consistent with the notions that sleep reduces cellular metabolic stress and that highly active organs may invoke distinct responses in this context. Implicating the UPRs in mitigating consequences of worm sleep loss indicates that these responses are deeply conserved.

## Methods

### Strains

Wild-type, transgenic, and mutant *C. elegans* strains were cultivated with OP50 bacteria according to standard protocols at 20 °C. The following strains were used: N2 Bristol (wild type), CF1038 *daf-16(mu86)*, GR1307 *daf-16(mgDf50)*, NQ116 *muIs211 [pNL213(ges-1p::GFP::daf-16) + rol-6(su1006)]* (gift from D. Raizen), NQ441 *daf-16(mgDf50); qnIs45[Pdaf-16:GFP::daf-16; Pmyo-2:mCherry]* (gift from D. Raizen at the University of Pennsylvania), SJ4151 *zcIs19 [ubl-5p::ubl-5::gfp]*, SJ4100 *zcIs13 [hsp-6p::gfp]*, SJ4058 *zcIs9 [hsp-60p::GFP + lin-15(+)]*, VC2564 *ubl-5(ok3389)*, SJ4200 *zcIs41 [ubl-5p::3xmyc-His tag::ubl-5 + myo-3p::gfp*]; SJ4151 *zcIs19 [ubl-5p::ubl-5::gfp]*, NQ128 *muEx169[unc-119p::GFP::daf-16 + rol-6(su1006)]* (gift from D. Raizen at the University of Pennsylvania), DA184 *ser-1(ok345), Is[Pmec-10::deg-3(u662)]* (gift from M. Treinin at the Hebrew University of Jerusalem), MT3002 *ced-3(n1286)*, MD701 *bcIs39 [lim-7p::ced-1::GFP + lin-15(+)]*, SJ4005 *zcIs4 [hsp-4p::gfp]*, RE666 *ire-1(v33)*, SJ17 *xbp-1(zc12)*, SJ30 *ire-1(zc14) II; zcIs4 V*, SJ17 *xbp-1(zc12) III; zcIs4 V*, RB925 *ire-1(ok799),* VC1099 *hsp-4(gk514)II*, UX564 *jnSi118[Pcomp-1::GFP::H2B::3’comp-1; Cb-unc-119(+)]; him-5(ok1896)* (gift from G. Stanfield at the University of Utah), LX1938 *egl-1(n986dm)V; vsIs164 X; lite-1(ce314) X; lin-15(n765ts) X* (gift from K. Collins at the University of Miami), LX1918 vsIs164 X; lite-1(ce314) X; lin-15(n765ts) X (gift from K. Collins at the University of Miami), IV205 ueEx122 [*str-3::TeTx::GFP*; *elt-2::sl2GFP*] (gift from S. Chalasani at the Salk Institute for Biological Studies).

### Severe nonlethal deprivation protocol

Motion and quiescence were identified using the image difference method as described in [129]. To disrupt quiescence during lethargus, mid-L4 animals were transferred to 60-mm nematode growth media (NGM) plates containing 10 mL of medium and seeded with 50 μL of OP50 culture that was grown overnight at 37 °C. Vibrations (1 kHz) were delivered using the mechanical clamps described in [11]. In contrast to the brief and well-spaced disruptions described previously, the stimuli described here were composed of 3 min of vibrations interspersed with 3 min of “quiet” intervals, i.e., a period of 6 min and a duty cycle of 50%.

Synchronization was performed in two steps. Gravid adults were allowed to lay eggs on a fresh plate for 2 h. Of the resulting larvae, mid and early L4s were manually picked for the deprived and control groups, respectively. Manually picking the L4 larvae reduced the variability of the timing of lethargus onset to 2 h.

Assays described throughout the manuscript employ one of three deprivation protocols: (1) fluorescent markers were assayed in individual worms during the first half of lethargus following a 1-h period of 3-min on/off disruptions. Control animals were either not stimulated at all (“mock”) or stimulated for an hour prior to lethargus. (2) Gene expression was assayed in small groups of tightly synchronized animals. Deprived groups were exposed to 3-min on/off disruptions for a period of 4 h that included lethargus, and control groups were exposed to 4 h of vibrations outside of lethargus. (3) Lasting defects were assayed in large groups of animals following a 12-h period of 3-min on/off stimulation. The stimulation period was initiated prior to lethargus and terminated at the young adult stage. Control animals were exposed to vibrations 5 h before lethargus, not stimulated for 5 h that included lethargus, and stimulated again for 5 h after lethargus. In cases when animals that were never stimulated were assayed, they were labeled “unperturbed” to differentiate them from the standard “control” groups.

### Optical measurements of pharyngeal pumping

Post-stimulus (see above) young adults were picked into liquid NGM and loaded into a WormSpa microfluidic device [65, 130]. An *Escherichia coli* OP50 overnight culture, concentration-adjusted in NGM to OD_600_ = 2.5 (an intermediate food concentration), was flown through the device at a constant rate throughout the assay. After 1 h of acclimation in the device, the animals were imaged for an additional 1 h at a magnification of 10× and 62.5 frames per second using a Basler acA1920-25um complementary metal-oxide-semiconductor (CMOS) camera mounted on Celestron 44104 microscopes [131]. Pumping events were identified using a custom Python script which aligned and subtracted consecutive images and calculated the entropy of the difference image. A pumping event resulted in a characteristic spike in this entropy [65, 132].

### Electropharyngeograms (EPGs)

Worms were loaded to a NemaMetrix ScreenChip System microfluidic device (NemaMetrix, Inc., Eugene, OR, USA) on a standard dissection microscope and allowed to acclimate for at least 30 min before recording. The device was filled with either bacterial food (OD_600_ = 2.5) or a solution of 10 mM 5-HT in NGM buffer. Immediately before the onset of the measurement, the output tube was disconnected to reduce electromagnetic noise. The pumping frequency was measured as described in the ScreenChip User Guide, and each recording lasted 5–10 min (measurements that terminated prematurely were discarded).

### Imaging of green fluorescent protein (GFP) reporters

The 1-h deprivation protocol consisted of 3-min-long vibration pulses interspersed with 3-min-long pauses, starting during the first 30 min of L4 lethargus and lasting a total of 1 h. Animals expressing a fluorescent reporter were exposed to these mechanical stimuli in artificial dirt microfluidic devices placed inside a standard petri dish. They were imaged in the device immediately before and after the 1-h period of disruption. Imaging was performed at a magnification of 20× (0.5 numerical aperture, NA) using a Nikon Eclipse Ti microscope (Nikon Inc., Melville, NY, USA) and an Andor iXon X3 EMCCD camera (Andor, Belfast, UK). Fluorescence intensity was determined using custom Python scripts.

### Calcium imaging in freely behaving animals

To reduce background fluorescence, calcium imaging was performed in artificial dirt microfluidic devices [133]. Animals co-expressing GCaMP5 and mCherry in their vms were mounted in the presence of bacterial food on an epi-fluorescence Nikon Eclipse Ti inverted microscope. Each worm was imaged at a magnification of 20× (0.5 NA) and a frame rate of 6 frames per second. Images were captured with an Andor iXon X3 EMCCD camera. A Dual View (DV2) two-channel system was used for simultaneous imaging of the red and green channels (Photometrix, Tucson, AZ, USA). Each animal was tracked manually and continuously imaged for a total period of 30 min. Calcium transients were analyzed using custom Matlab scripts (The Mathworks Inc., Natick, MA, USA).

### Brood size

Brood size was counted by visual inspection: 10 h after mechanical stimulation ended, animals were transferred to individual 60-mm NGM plates seeded with a 50-μL drop of OP50 (two animals per plate). Plates were scored in the morning and evening of the following 3 days. To avoid the accumulation of hatched offspring, animals were transferred to new plates prior to the evening counts each day. For male mating, two males and two hermaphrodites were transferred to each plate.

### Sperm counting

Adult *Pcomp-1::GFP::H2B::3’comp-1* animals 24 h post-L4 lethargus were sealed into individual “artificial dirt” chambers filled with NGM and 10 μM levamisole. Confocal images of fluorescently labeled sperm were obtained using a Zeiss LSM 880 microscope with a Plan-Apochromat 40×/1.4 oil differential interference contrast (DIC) objective. Image stacks were analyzed using the FARSIGHT Nucleus Editor (http://www.farsight-toolkit.org/wiki/NucleusEditor). We note that when the fluorescent marker was crossed to a *daf-16* mutant background, it was not confined to the nuclei. However, individual sperm cells were still detectable. We observed this in all *daf-16(mgDf60)* mutants and 25% of the *daf-16(mu86)* mutants.

### Real-time PCR

The *lmn-1* gene, encoding the *C. elegans* nuclear laminin, was chosen as the endogenous control gene. Primers (except for *lmn-1*, Shaham lab, Rockefeller University, private communication) were designed using Wormbase.org and the National Center for Biotechnology Information (NCBI) Primer-Basic Local Alignment Search Tool (BLAST) software. They were tested for specificity using NCBI BLAST and by agarose gel electrophoresis (using genomic DNA) and purchased from Integrated DNA Technologies (IDT, Coralville, IA, USA). The primers used for *lmn-1*, *hsp-4*, *ubl-5*, and *hsp-6* were TCGAGGCGGAAAAGGCTC (Fwd), GCTCCAGCGAGTTCTCTCTC (Rev), GCCGACAAGGAAAAACTCGG (Fwd), GTGGGGTTGGGTTGGGAAA (Rev), ACAAACTGGAACACGATGGGA (Fwd), TCCCTCGTGAATCTCGTAATCC (Rev), AAGAACTCTGGAGGTGACGC (Fwd), and ACGTTGGGGGTTTCTAAAGAT (Rev), respectively. Real-time quantitative PCR amplifications were performed using 25 μL of QuantiTect SYBR Green Real-Time PCR master mix (QIAGEN, Hilden, Germany), 2 μL of diluted reverse transcription product (2 ng/reaction), 1.5 μL each of forward and reverse primer, and 20 μL of DNase/RNase-free water in a total volume of 50 μL. Amplification was carried out in an AB 7900 HT Real-Time PCR cycler (Applied Biosystems, Foster City, CA, USA) with initial polymerase activation at 95 °C for 15 min, followed by cycles of 94 °C for 15 s denaturation, 57 °C for 30 s for primer-specific annealing, and 72 °C for 30 s for extension. A melting curve analysis was carried out (60–95 °C) to verify the specificity of amplicons, i.e., the absence of primer dimers and nonspecific products. Each assay included six technical replicates and a no-template control for every primer pair.

### Statistical analysis

Pairwise comparisons of data represented in bar or box plot were done using the Student’s *t* test. In the case of multiple comparisons, significance was calculated using a one-way analysis of variance (ANOVA) test and the Bonferroni post hoc correction. Distributions represented by histograms were compared using the *k*-sample Anderson-Darling test and the Bonferroni post hoc correction for multiple comparisons (when applicable).

For each figure panel, the following *p* values are listed in order of the positions of asterisks and not significant (n.s.) labels (top to bottom and then left to right). Fig. 1b: 1.1 × 10^–3^, 6.5 × 10^–15^, 2.2 × 10^–15^, 9.5 × 10^–7^. Fig. 1c: 0.59, 0.84, 0.002, 0.001. Fig. 2b: 0.003, 0.41, 0.63. Fig. 2c: 4.9 × 10^–5^. Fig. 2d: 0.36, 0.98, 0.033, 0.164. Fig. 3a: 0.016, 0.11, 0.34. Fig. 3b: 0.04, 0.22. Fig. 3c: 0.003, 0.001, 0.005, 0.002. Fig. 4a: 0.21, 0.048, 0.010, 0.50, 0.26, 0.46. Fig. 4b: 0.006, 0.40, 0.142, 0.074, 0.42. Fig. 5a: 0.018, 0.095, 0.38. Fig. 5b: 0.50. Fig. 6a: 4.8 × 10^–3^_._ Fig. 6b: 5.4 × 10^–14^. Fig. 6c: 6.1 × 10^–3^. Fig. 6d: 0.39. Fig. 6e: 0.33. Fig. 6f: 0.007. Fig. 6h: 0.19. Fig. 6i: 0.48. Fig. 7a: 0.46. 0.18. Fig. 7b: 0.009, 0.44. Fig. 7c: 0.050, 0.043, 0.009, 0.008. Fig. 8c: 0.38, 0.017, 0.79. Additional file 1: Figure S1: 0.43. Additional file 3: Figure S3A: 0.37, 0.44, 0.003, 0.50, 0.34, 0.41. Additional file 3: Figure S3B: 0.048, 0.028, 0.40, 0.070, 0.27, 0.50. Additional file 5: Figure S5: 0.16, 0.050, 0.010. Additional file 6: Figure S6B: 0.035, 0.042, 0.021. Additional file 6: Figure S6C: 0.002. Additional file 6: Figure S6D: 0.061, 0.005, 0.28. Additional file 7: Figure S7 (OD_600_ = 2.5): 0.15, 0.37, 0.22, 0.34, 0.092, 0.36, 0.30, 0.30. Additional file 7: Figure S7 (10 mM 5-HT): 0.039, 0.043, 0.22, 0.47, 0.27, 0.30. Additional file 8: Figure S8A: 2.5 × 10^–4^, 0.82. Additional file 8: Figure S8B: 0.77. Additional file 10: Figure S10: 0.10, 0.34, 0.26, 0.29.

## Additional files

Additional file 1: Figure S1. Fluorescence of the *hsp-60p::GFP* fluorescent reporter before and after deprivation. In our hands, elevated expression of the reporter was not observed after 1 h of disrupting worm sleep. (PDF 368 kb)

Additional file 2: Figure S2. Pumping rates increase as a function of food availability. The average pumping rate of wild-type animals at different concentrations of ambient bacterial food (as measured by optical density, *OD*_*600*_). *N* = 10 animals per condition. Error bars depict mean ± standard error of the mean (SEM). (PDF 318 kb)

Additional file 3: Figure S3. Duty ratios of rapid pumping of deprived and control worms. (A) Box plots of duty ratios for control and deprived animals. Continuous pumping was defined as a period in which the delay between pumps did not exceed 500 ms. (B) Same as (A) for UPR mutants and the neuronal rescue of *daf-16*. (PDF 1224 kb)

Additional file 4: Figure S4. Genetic ablation of the *mec-10* expressing touch neurons abolishes responses to the vibration stimuli. Locomotion in response to 1-kHz vibrations was quantified as the fraction of the body area the animal traversed per second. Wild-type animals (*left*) responded robustly to the stimulus, while touch-insensitive *mec-10p::deg-3(gf)* transgenics (*right*) did not exhibit a detectable response. *N* = 20 animals from each genotype were assayed, and *shaded areas* depict mean ± SEM. (PDF 468 kb)

Additional file 5: Figure S5. EPG measurements of pumping fatigue are consistent with the results of the optical tracking method. Instantaneous pumping rates were calculated as 1/(duration between consecutive contraction peaks). Mean (per animal) rates for wild-type animals, *daf-16(mu86)* mutants, and *ubl-5(ok3389)* mutants reproduced the phenotypes detected by optical tracking. (PDF 480 kb)

Additional file 6: Figure S6. The duration of an individual pumping motion is extended by exposure to mechanical vibrations irrespective of the timing of the stimuli. (A) *Top*: a sample EPG trace of a wild-type animal in the presence of food at OD_600_ = 2.5 concentration. Peaks correspond to corpus and terminal bulb contraction. Troughs correspond to corpus relaxation. *Bottom*: average contraction and relaxation EPG traces for undisrupted and deprived wild-type animals. Distributions of pump durations are shown for undisrupted, deprived, and control (exposed to vibrations outside of lethargus) animals. The outline of the distribution for undisrupted animals was duplicated as a guide to the eye. (B) The mean (per animal) pump durations and amplitudes of EPG peaks and troughs for wild-type animals (*top*) and *daf-16(mu86)* mutants (*bottom*). (C) Same as (A, B) for UPR^mt^ deficient *ubl-5* mutants. (D) Same as (A, B) for mutants treated with 10 mM 5-HT instead of food. *Horizontal lines*, *boxes*, and *bars* depict medians, 1st and 3rd quartiles, and 5th and 95th percentiles, respectively. Sample sizes are noted in parentheses; *asterisks* and *double asterisks* denote significant differences (*p* < 0.05 and *p* < 0.01, respectively). (PDF 774 kb)

Additional file 7: Figure S7. EPG characteristics of individual pumping motions. Mean (per animal) EPG peak and trough amplitudes for animals presented with food or 10 mM 5-HT. Vibration stimuli during or outside lethargus did not typically affect these amplitudes. Sample sizes are noted in parentheses, and *asterisks* denote significant differences (*p* < 0.05). (PDF 1006 kb)

Additional file 8: Figure S8. (A) Brood size can be reduced by sleep depriving either males or hermaphrodites. The numbers of egg laid by *daf-16(mgDf50)* mutants that mated with males. Either the males, the hermaphrodites, or both were sleep deprived. (B) ASI neurons are required for sleep deprivation to impact fecundity. Brood size was not reduced by deprivation when ASI neurons were genetically ablated using tetanus toxin (in contrast to phenotypes shown in Fig. 6a–c, f). (PDF 176 kb)

Additional file 9: Figure S9. Nonlethal sleep deprivation does not increase egg retention. The numbers of fertilized eggs retained in the uterus of wild-type animals and *daf-16* mutants 24 h and 48 h after L4 lethargus. Deprived animals were exposed to the stimulus before, during, and after L4 lethargus. Control animals were exposed to the stimulus before and after L4 lethargus. (PDF 381 kb)

Additional file 10: Figure S10. Mean velocities are not affected by deprivation. No significant differences were found between mean velocities (averaged over the 30 min of the assay) of undisrupted or deprived wild-type animals, *ire-1* mutants, or *ubl-5* mutants. *Horizontal lines*, *boxes*, and *bars* depict medians, 1st and 3rd quartiles, and 5th and 95th percentiles, respectively. Sample sizes are noted in parentheses. (PDF 564 kb)

Additional file 11: Figure S11. Long-term mean velocities with vm twitching. Mean velocities and vm twitching were significantly correlated in wild-type animals, *ubl-5* mutants, and undisrupted *ire-1* mutants. Notably, these correlations were stronger in sleep-deprived wild-type animals and *ubl-5* mutants as compared to undisrupted worms of the corresponding genotype. However, correlations did not increase in *ire-1* mutants, suggesting that secreted proteins may be required for deprivation-induced enhancement of coordination between vm activity and locomotion. (PDF 481 kb)
