# Supplementary figures and images for: Distinct unfolded protein responses mitigate or mediate effects of nonlethal deprivation of C. elegans sleep in different tissues
Source: BMC Biol. 2017 Aug 28;15:67. doi: 10.1186/s12915-017-0407-1 (PMC5572162; doi:10.1186/s12915-017-0407-1)

Figure S1 - Sanders et al.

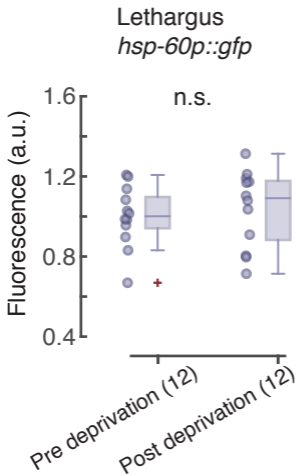

Supplement: Supplementary file 1 — Fluorescence of the hsp-60p::GFP fluorescent reporter before and after deprivation. In our hands, elevated expression of the reporter was not observed after 1 h of disrupting worm sleep. (PDF 368 kb) [file 12915_2017_407_MOESM1_ESM.pdf]

Figure S2 - Sanders et al.

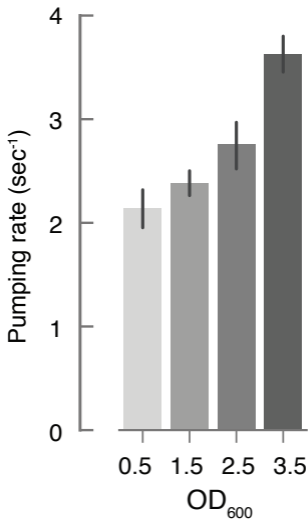

Supplement: Supplementary file 2 — Pumping rates increase as a function of food availability. The average pumping rate of wild-type animals at different concentrations of ambient bacterial food (as measured by optical density, OD 600). N = 10 animals per condition. Error bars depict mean ± standard error of the mean (SEM). (PDF 318 kb) [file 12915_2017_407_MOESM2_ESM.pdf]

Figure S3 - Sanders et al.

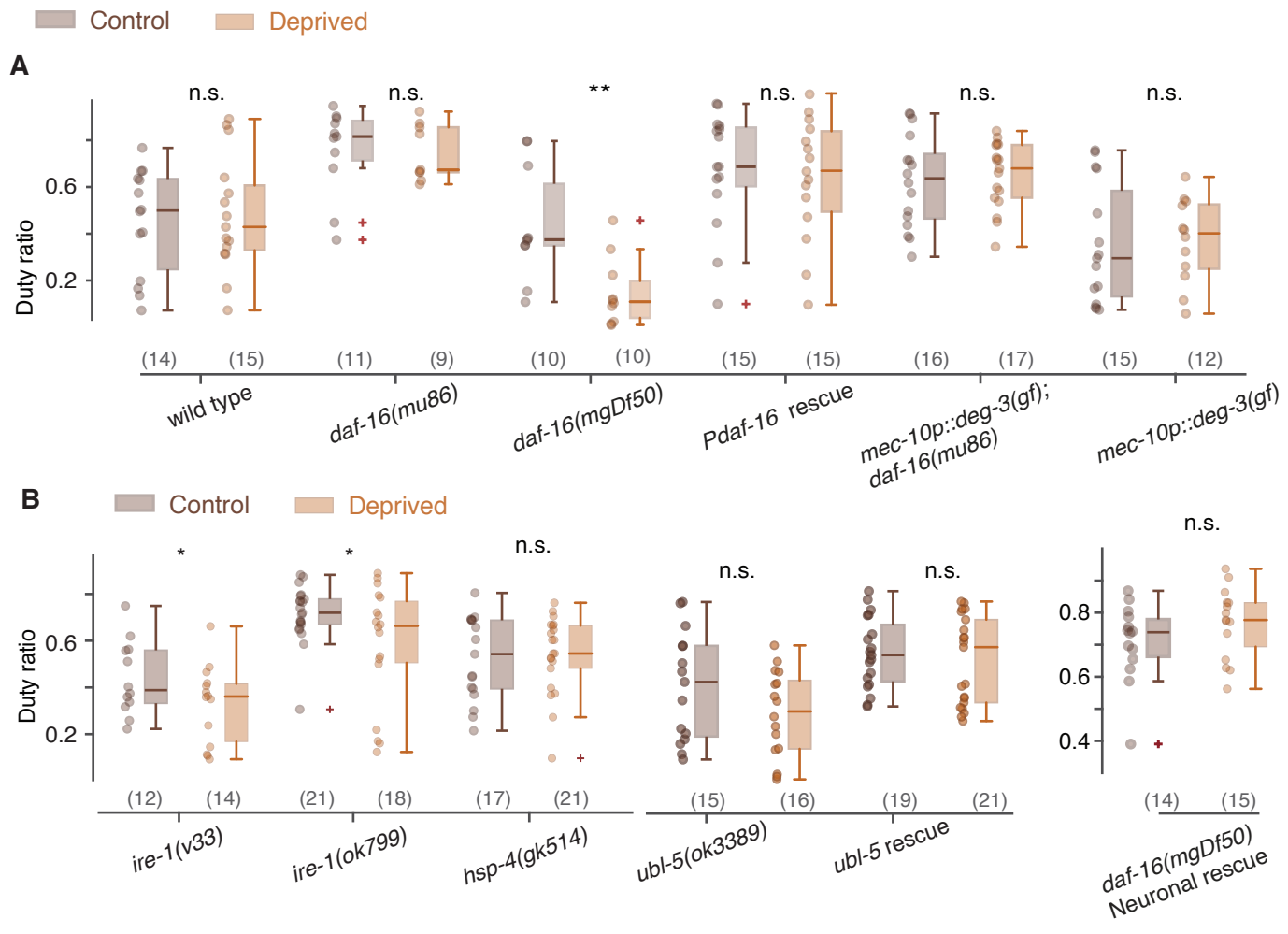

Supplement: Supplementary file 3 — Duty ratios of rapid pumping of deprived and control worms. (A) Box plots of duty ratios for control and deprived animals. Continuous pumping was defined as a period in which the delay between pumps did not exceed 500 ms. (B) Same as (A) for UPR mutants and the neuronal rescue of daf-16. (PDF 1224 kb) [file 12915_2017_407_MOESM3_ESM.pdf]

Figure S4 - Sanders et al.

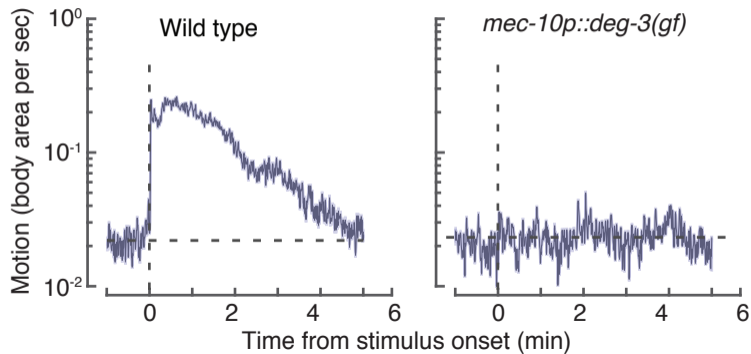

Supplement: Supplementary file 4 — Genetic ablation of the mec-10 expressing touch neurons abolishes responses to the vibration stimuli. Locomotion in response to 1-kHz vibrations was quantified as the fraction of the body area the animal traversed per second. Wild-type animals (left) responded robustly to the stimulus, while touch-insensitive mec-10p::deg-3(gf) transgenics (right) did not exhibit a detectable response. N = 20 animals from each genotype were assayed, and shaded areas depict mean ± SEM. (PDF 468 kb) [file 12915_2017_407_MOESM4_ESM.pdf]

Figure S5 - Sanders et al.

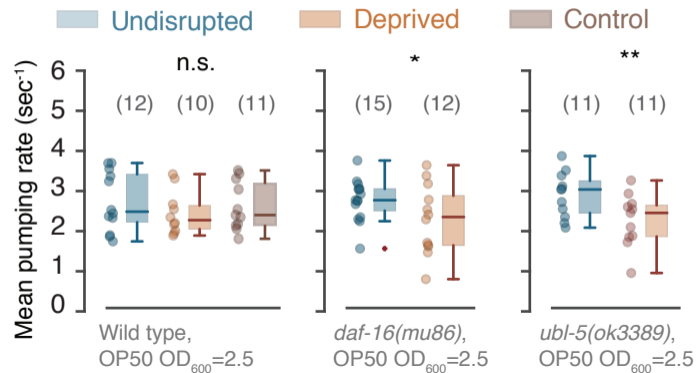

Supplement: Supplementary file 5 — EPG measurements of pumping fatigue are consistent with the results of the optical tracking method. Instantaneous pumping rates were calculated as 1/(duration between consecutive contraction peaks). Mean (per animal) rates for wild-type animals, daf-16(mu86) mutants, and ubl-5(ok3389) mutants reproduced the phenotypes detected by optical tracking. (PDF 480 kb) [file 12915_2017_407_MOESM5_ESM.pdf]

Figure S6 - Sanders et al.

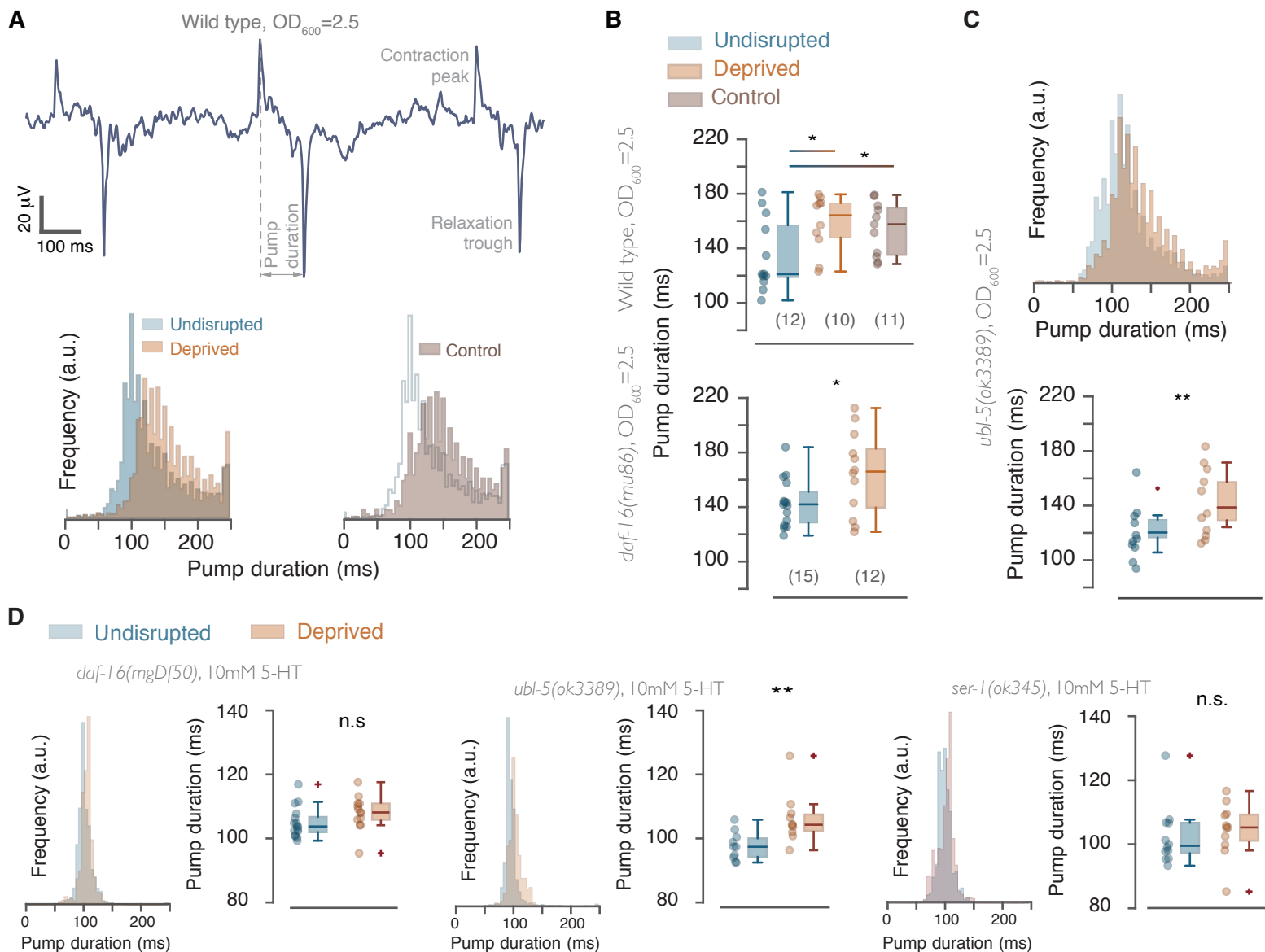

Supplement: Supplementary file 6 — The duration of an individual pumping motion is extended by exposure to mechanical vibrations irrespective of the timing of the stimuli. (A) Top: a sample EPG trace of a wild-type animal in the presence of food at OD600 = 2.5 concentration. Peaks correspond to corpus and terminal bulb contraction. Troughs correspond to corpus relaxation. Bottom: average contraction and relaxation EPG traces for undisrupted and deprived wild-type animals. Distributions of pump durations are shown for undisrupted, deprived, and control (exposed to vibrations outside of lethargus) animals. The outline of the distribution for undisrupted animals was duplicated as a guide to the eye. (B) The mean (per animal) pump durations and amplitudes of EPG peaks and troughs for wild-type animals (top) and daf-16(mu86) mutants (bottom). (C) Same as (A, B) for UPRmt deficient ubl-5 mutants. (D) Same as (A, B) for mutants treated with 10 mM 5-HT instead of food. Horizontal lines, boxes, and bars depict medians, 1st and 3rd quartiles, and 5th and 95th percentiles, respectively. Sample sizes are noted in parentheses; asterisks and double asterisks denote significant differences (p < 0.05 and p < 0.01, respectively). (PDF 774 kb) [file 12915_2017_407_MOESM6_ESM.pdf]

Figure S7 - Sanders et al.

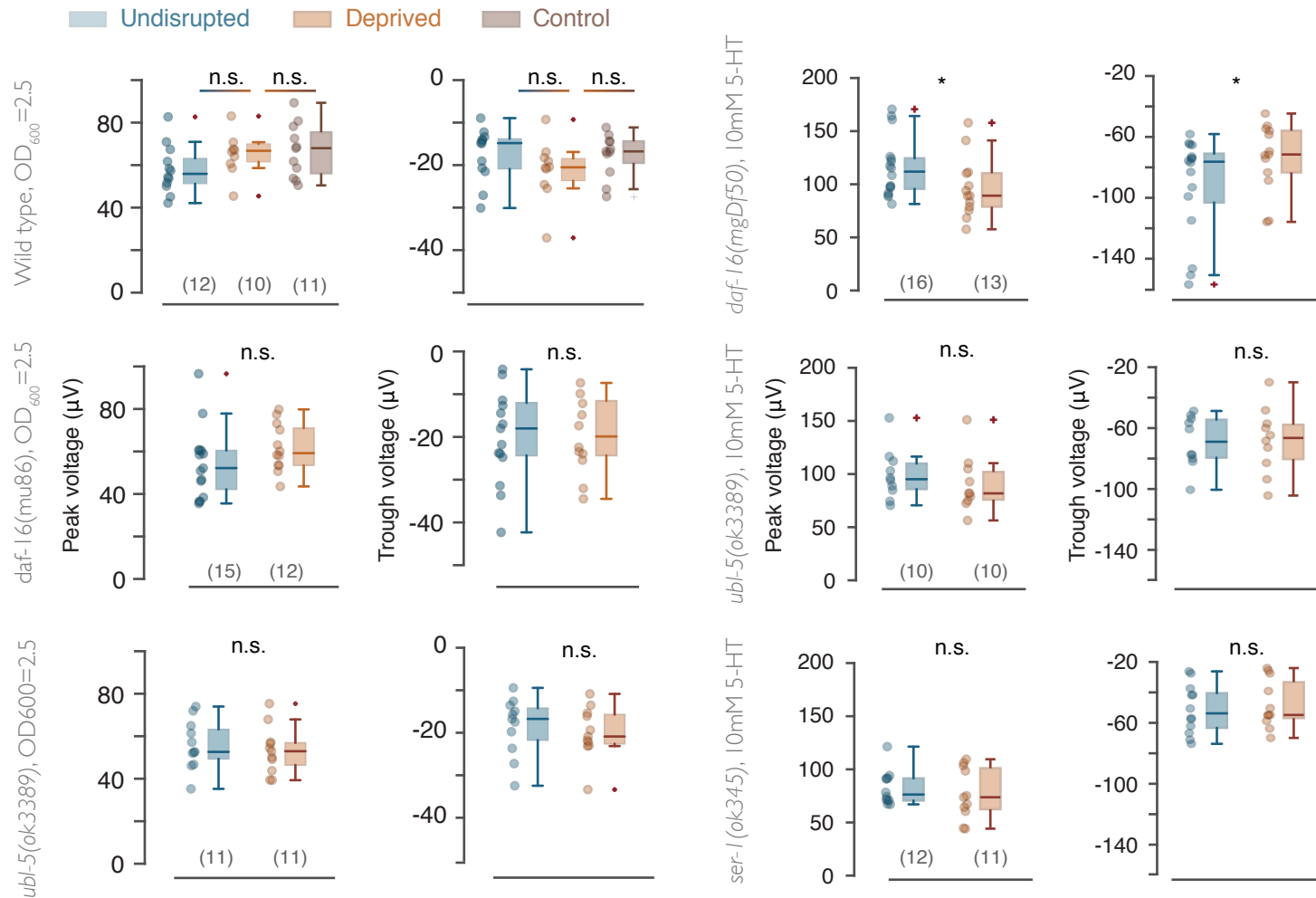

Supplement: Supplementary file 7 — EPG characteristics of individual pumping motions. Mean (per animal) EPG peak and trough amplitudes for animals presented with food or 10 mM 5-HT. Vibration stimuli during or outside lethargus did not typically affect these amplitudes. Sample sizes are noted in parentheses, and asterisks denote significant differences (p < 0.05). (PDF 1006 kb) [file 12915_2017_407_MOESM7_ESM.pdf]

Figure S8 - Sanders et al.

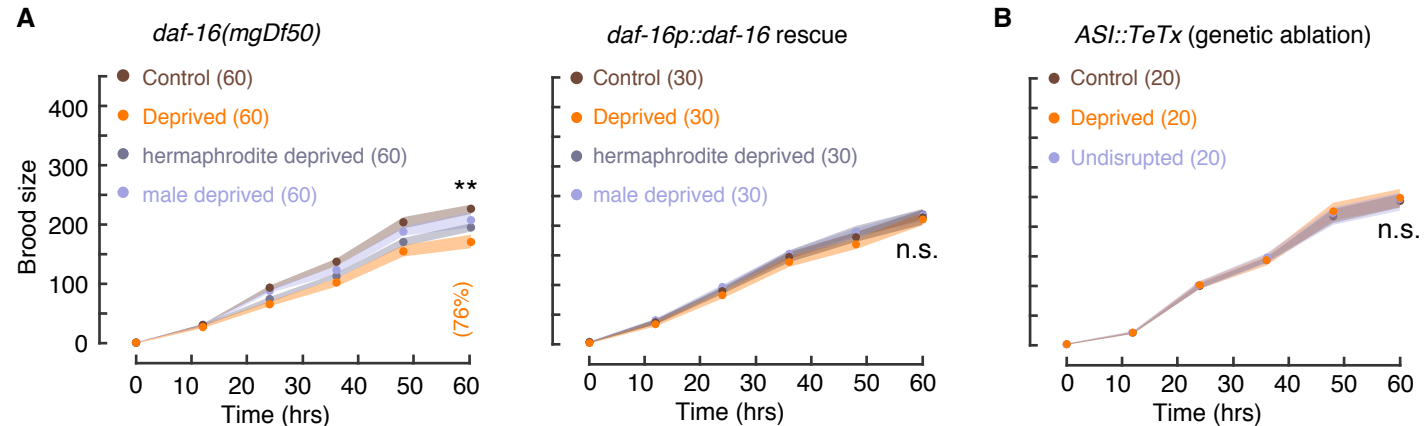

Supplement: Supplementary file 8 — (A) Brood size can be reduced by sleep depriving either males or hermaphrodites. The numbers of egg laid by daf-16(mgDf50) mutants that mated with males. Either the males, the hermaphrodites, or both were sleep deprived. (B) ASI neurons are required for sleep deprivation to impact fecundity. Brood size was not reduced by deprivation when ASI neurons were genetically ablated using tetanus toxin (in contrast to phenotypes shown in Fig. 6a–c, f). (PDF 176 kb) [file 12915_2017_407_MOESM8_ESM.pdf]

Figure S9 - Sanders et al.

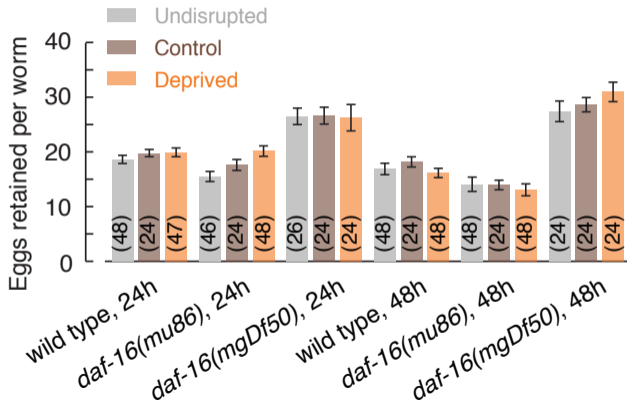

Supplement: Supplementary file 9 — Nonlethal sleep deprivation does not increase egg retention. The numbers of fertilized eggs retained in the uterus of wild-type animals and daf-16 mutants 24 h and 48 h after L4 lethargus. Deprived animals were exposed to the stimulus before, during, and after L4 lethargus. Control animals were exposed to the stimulus before and after L4 lethargus. (PDF 381 kb) [file 12915_2017_407_MOESM9_ESM.pdf]

Figure S10 - Sanders et al.

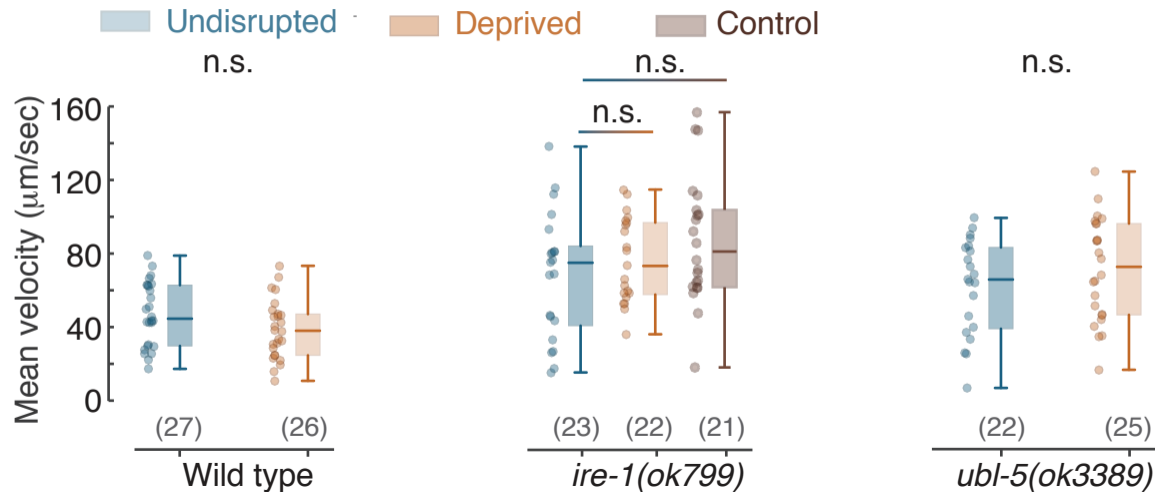

Supplement: Supplementary file 10 — Mean velocities are not affected by deprivation. No significant differences were found between mean velocities (averaged over the 30 min of the assay) of undisrupted or deprived wild-type animals, ire-1 mutants, or ubl-5 mutants. Horizontal lines, boxes, and bars depict medians, 1st and 3rd quartiles, and 5th and 95th percentiles, respectively. Sample sizes are noted in parentheses. (PDF 564 kb) [file 12915_2017_407_MOESM10_ESM.pdf]

Figure S11 - Sanders et al.

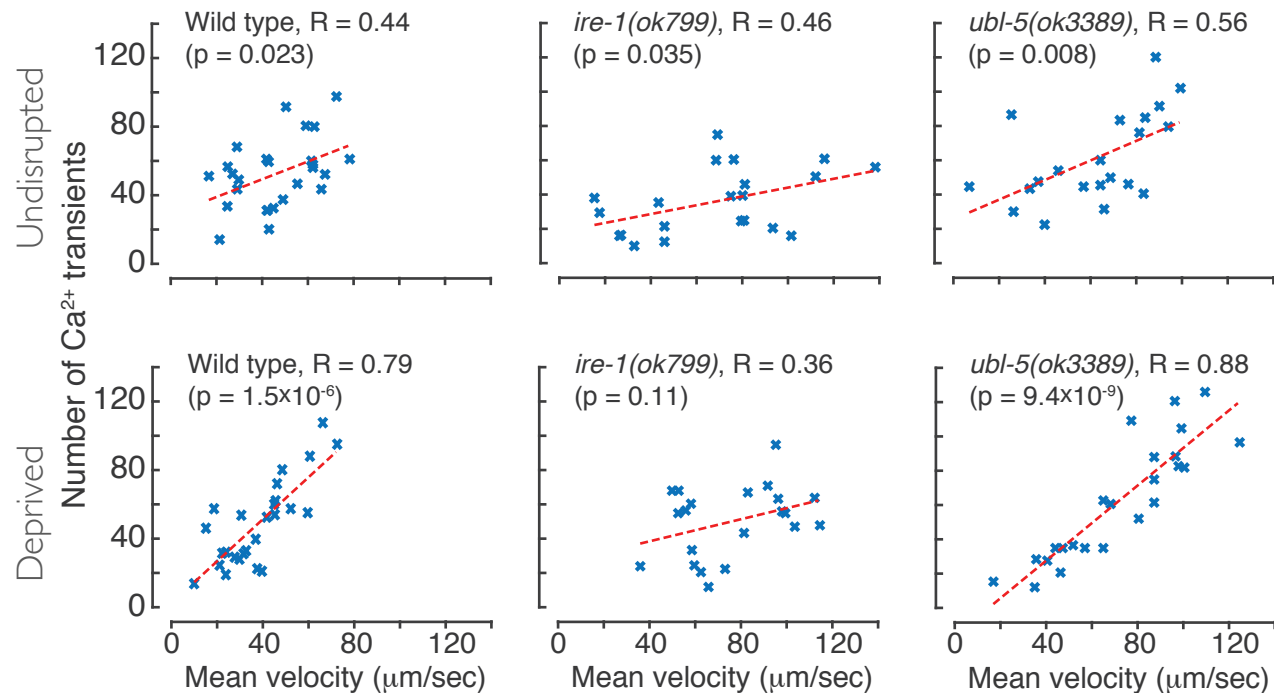

Supplement: Supplementary file 11 — Long-term mean velocities with vm twitching. Mean velocities and vm twitching were significantly correlated in wild-type animals, ubl-5 mutants, and undisrupted ire-1 mutants. Notably, these correlations were stronger in sleep-deprived wild-type animals and ubl-5 mutants as compared to undisrupted worms of the corresponding genotype. However, correlations did not increase in ire-1 mutants, suggesting that secreted proteins may be required for deprivation-induced enhancement of coordination between vm activity and locomotion. (PDF 481 kb) [file 12915_2017_407_MOESM11_ESM.pdf]
